# Clinical characteristics, management strategies, and survival outcomes of patients with chronic thromboembolic pulmonary hypertension in Central Asia: experience from the sole pulmonary endarterectomy center

**DOI:** 10.3389/fcvm.2026.1786958

**Published:** 2026-03-24

**Authors:** Anara Abbay, Akbota Askanbekova, Yuliya Semenova, Aigerim Kuzhakhmetova, Gulzhamal Duysenbay, Murat Mukarov, Timur Lesbekov

**Affiliations:** 1 Department of Medicine, Nazarbayev University School of Medicine, Astana, Kazakhstan; 2 Department of Cardiac Surgery, Heart Center, University Medical Center, Astana, Kazakhstan

**Keywords:** chronic thromboembolic pulmonary hypertension, pulmonary endarterectomy, pulmonary hypertension, CTEPH registry, Kazakhstan, survival

## Abstract

**Background:**

Chronic thromboembolic pulmonary hypertension (CTEPH) is a potentially curable cause of pulmonary hypertension, yet data from Central Asia, including Kazakhstan, remain scarce. We aimed to characterize real-world management strategies and outcomes of patients with CTEPH treated within a national referral program.

**Methods:**

We conducted a retrospective analysis of consecutive adult patients diagnosed with CTEPH between 2018 and 2024 at the sole national CTEPH referral center in Kazakhstan. Clinical characteristics, imaging findings, and invasive hemodynamic data were compared between patients undergoing pulmonary endarterectomy (PEA) and those receiving medical therapy alone. Pre- and post-operative changes were assessed in a subgroup of surgical patients, and long-term survival was evaluated.

**Results:**

A total of 110 patients were included (mean age 54 ± 12 years; 58% male), of whom 56 (50.9%) underwent PEA. The median delay from symptom onset to referral was 24 months. No significant differences in baseline hemodynamic parameters were observed between surgical and medical groups. Among patients with available paired data, PEA was associated with substantial improvements in pulmonary hemodynamics, right-heart function, exercise capacity, and biomarkers of cardiac stress. Over a median follow-up of 39 months, survival was numerically higher in the surgical group, although this difference did not reach statistical significance. The estimated nationally diagnosed catheter-confirmed CTEPH detection rate was low, whereas the annual number of PEA procedures increased over time.

**Conclusion:**

Outcomes of PEA in Kazakhstan fall within the ranges reported in international surgical series. These findings highlight the need for earlier diagnosis, structured post-pulmonary embolism surveillance, and centralized multidisciplinary care to improve access to curative treatment in emerging healthcare systems. The observed nationally diagnosed detection rates likely underestimate the true population burden of CTEPH.

## Introduction

Chronic thromboembolic pulmonary hypertension (CTEPH) is a distinct form of pulmonary hypertension resulting from incomplete resolution of pulmonary emboli and subsequent remodeling of the pulmonary vasculature. The disease is characterized by progressive dyspnea, exercise limitation, and right-ventricular failure, and is classified as Group 4 pulmonary hypertension according to current international guidelines (1). Without timely recognition and treatment, CTEPH is associated with substantial morbidity and mortality (2).

Pulmonary endarterectomy (PEA) remains the treatment of choice for operable CTEPH and offers the potential for cure, with excellent long-term survival and sustained hemodynamic improvement when performed in experienced centers (3). For patients with surgically inaccessible disease or persistent pulmonary hypertension after surgery, balloon pulmonary angioplasty (BPA) and targeted medical therapy, most notably soluble guanylate cyclase stimulation, form part of a contemporary multimodal treatment strategy (4, 5). Current European guidelines emphasize lifelong anticoagulation, centralized operability assessment, and individualized sequencing of interventional and pharmacologic therapies (1).

Despite these advances, CTEPH remains underdiagnosed worldwide. National registry data indicate that the annual incidence rates range from 3 to 6 cases per million adults, largely reflecting differences in disease awareness, referral patterns, and access to specialized centers (6). Delayed diagnosis remains common, with many patients referred more than a year after symptom onset, often at an advanced stage of disease (1).

Data from Central Asia are largely absent, as no published registries or population-based studies have been reported to date. Kazakhstan, a country with a population of approximately 20 million, hosts a single national center capable of performing PEA. The deficit of regional data limits understanding of disease burden, referral efficiency, and treatment outcomes, and hampers strategic planning for specialized pulmonary hypertension services.

To address this gap, we conducted a retrospective analysis of all patients diagnosed with CTEPH at the national referral center in Kazakhstan over a seven-year period. The objectives of this study were to characterize baseline clinical and hemodynamic features, compare outcomes between surgical and medical management strategies, assess long-term survival, and estimate national trends in CTEPH detection and PEA utilization.

## Methods

### Study design and population

This observational, retrospective, single-center study was conducted at a national referral center performing PEA in Kazakhstan. Discharge summaries of adult patients (≥18 years) with confirmed CTEPH (ICD-10 I27.24) admitted between January 1, 2018, and December 31, 2024, were screened. Patients were included if the diagnosis was established by right-heart catheterization, defined by a mean pulmonary artery pressure >20 mmHg, pulmonary vascular resistance >2 Wood units, and pulmonary artery wedge pressure ≤15 mmHg, together with radiologic evidence of chronic pulmonary artery obstruction, in accordance with contemporary ESC/ERS guideline definitions (1). Among 173 hospitalizations initially identified, repeat admissions were excluded, yielding 110 unique patients with imaging- and right-heart catheterization–confirmed CTEPH. Patients were categorized by management strategy (PEA vs. medical therapy alone). Operability was determined by a multidisciplinary team including pulmonary hypertension cardiologists and experienced cardiothoracic surgeons. Decisions were based primarily on the anatomical distribution and surgical accessibility of thromboembolic lesions on pulmonary angiography and/or CT pulmonary angiography, comorbidity profile, and perceived operative risk. Surgical level classification (San Diego/Jamieson classification) was available for patients undergoing surgical management, but was not systematically recorded for the entire cohort due to retrospective design. At the time of the study, balloon pulmonary angioplasty was not available in Kazakhstan. Patient selection is summarized in Supplementary Figure S1.

### Study design and data collection

The study was conducted in three predefined analytical stages. Baseline characterization included the collection of demographic characteristics, comorbidities, functional status, laboratory values, echocardiographic parameters, and invasive hemodynamic data for 110 unique patients at first admission to the pulmonary hypertension program. Annual nationally diagnosed catheter-confirmed CTEPH detection rates during the study period were calculated using mid-year population estimates obtained from the national statistical office.

Outcome analysis: survival status was assessed using hospital records and structured telephone interviews with patients or close relatives. Overall survival was analyzed for the entire cohort of uniquely identified patients.

Treatment effect analysis: In patients undergoing PEA, paired pre- and postoperative clinical, echocardiographic, and hemodynamic data were analyzed to evaluate dynamic treatment effects. Patients with available paired clinical and hemodynamic data and a follow-up interval of at least 3 months after PEA were included (*n* = 22). Follow-up assessments were performed during routine postoperative clinical evaluations.

Functional capacity was assessed using the six-minute walk distance. Echocardiographic measurements were performed according to guideline-recommended protocols, and right-heart catheterization was performed according to institutional standards. All assessments were performed in accordance with contemporary international guideline recommendations and institutional protocols.

### Statistical analysis

Statistical analysis was performed using Statistical Package for the Social Sciences (SPSS), version 27 (IBM Corp., Armonk, NY, USA). Continuous variables are presented as mean ± standard deviation or median with interquartile range, and categorical variables as counts and percentages. Due to the retrospective design, some clinical and hemodynamic variables were unavailable for all patients. Analyses were performed using the available-case approach, and no imputation of missing data was undertaken. The pattern of missingness was primarily attributable to incomplete historical documentation rather than systematic group differences. Between-group comparisons were conducted using Student's *t*-test or Mann–Whitney *U* test for continuous variables and *χ*² or Fisher's exact test for categorical variables. Paired comparisons in surgical patients were performed using the paired t-test. Survival was analyzed using the Kaplan–Meier method, and comparisons were assessed using the log-rank test. Univariable Cox proportional hazards regression was used to explore associations with mortality. The proportional hazards assumption was assessed graphically using log-minus-log survival plots. Given the limited number of events, multivariable modeling was not performed to avoid overfitting. A two-sided *p*-value < 0.05 was considered statistically significant.

## Results

### Study population and baseline characteristics

Between January 2018 and December 2024, 173 hospitalizations coded as CTEPH (ICD-10 I27.24) were identified. After exclusion of repeat admissions, 110 unique adult patients with imaging- and right-heart catheterization-confirmed CTEPH were included in the analysis. Of these, 56 (50.9%) underwent PEA, and 54 (49.1%) received medical management alone.

Baseline demographic, functional, and clinical characteristics are summarized in Table 1. The mean age of the cohort was 54.4 ± 12.2 years, and 58.2% were male. The median delay between symptom onset and referral to the pulmonary hypertension (PH) center was 24 months. Hospitalization duration was longer among surgical patients (18.9 ± 9.4 days) than among medically managed patients (8.3 ± 4.4 days; *p* < 0.001). Most patients presented with significant functional limitation, with 62.5% classified as WHO (World Health Organization) functional class III. Among patients with available San Diego classification (*n* = 92), proximal disease (levels I-II) was observed in 72.9%, whereas distal disease (levels III-IV) was observed in 27.2%, with a significant difference between treatment groups (*p* < 0.001). Pulmonary embolism (PE) was the most prevalent comorbidity (69.1%), followed by deep-vein thrombosis (56.4%), thrombophilia (28.2%), and varicose veins (17.3%). Varicose veins were more frequent among PEA patients than among medically managed patients (26.8% vs. 7.4%; *p* = 0.007).

**Table 1:** Baseline functional class, comorbidities, and clinical parameters (*n* = 110).

Variable	All patients (*N* = 110)	PEA patients (*N* = 56)	Medical management (*N* = 54)	*p*-value
Age, years, ±SD	54.38 ± 12.22	52.95 ± 12.29	55.87 ± 12.02	0.210
Gender, males, *n* (%)	64 (58.2%)	34 (60.7%)	30 (55.6%)	0.583
Time from dyspnea onset to referral to PH center, years, median [IQR]	2.0 (1.0–4.0)	2.0 (1.0–3.8)	2.0 (1.0–4.0)	0.549
Hospitalization duration, days, ±SD	13.64 ± 9.28	18.91 ± 9.41	8.26 ± 4.44	<0.001
San Diego class (*N* = 92)				<0.001
I	34 (37.0)	24 (43.6)	10 (27.0)	
II	33 (35.9)	25 (45.5)	8 (21.6)	
III	19 (20.7)	6 (10.9)	13 (35.1)	
IV	6 (6.5)	0 (0.0)	6 (16.2)	
WHO functional class, % (*N* = 102)				0.299
I	2 (1.9)	1 (1.9)	1 (2.0)	
II	16 (15.4)	5 (9.3)	11 (22.0)	
III	65 (62.5)	35 (64.8)	30 (60.0)	
IV	21 (20.2)	13 (24.1)	8 (16.0)	
Comorbidities				
Pulmonary embolism, *n* (%)	76 (69.1)	39 (69.6)	37 (68.5)	0.375
Deep vein thrombosis, *n* (%)	62 (56.4)	33 (58.9)	29 (53.7)	0.581
Varicose veins, *n* (%)	19 (17.3)	15 (26.8)	4 (7.4)	0.007
Thrombophilia, *n* (%)	31 (28.2)	20 (35.7)	11 (20.4)	0.074
Clinical parameters, mean ± SD				
6 min walking distance, m	265.0 ± 94.5	254.5 ± 115.5	274.1 ± 69.3	0.386
BMI, kg/m^2^	28.6 ± 5.5	27.1 ± 4.1	30.2 ± 6.4	0.004
NT-proBNP, pg/mL, median (IQR)	1,460 (900–4,020)	1,450 (818–4,082)	1,473 (977–3,969)	0.939

WHO, World Health Organization; PEA, pulmonary endarterectomy; BMI, body mass index; NT-proBNP, N-terminal pro–B-type natriuretic peptide.

The mean six-minute walk distance (6MWD) was 265 ± 95 m and did not differ significantly between groups. Mean body mass index (BMI) was 28.6 ± 5.5 kg/m^2^ and was higher in medically managed patients (30.2 ± 6.4 vs. 27.1 ± 4.1 kg/m²; *p* = 0.004). NT-proBNP levels did not differ significantly between treatment groups (*p* = 0.939).

Baseline echocardiographic and right-heart catheterization parameters are presented in Table 2. Mean right-ventricular systolic pressure (RVSP) was 73.4 ± 26.8 mmHg, mean pulmonary artery pressure (mPAP) was 40.5 ± 14.3 mmHg, and pulmonary vascular resistance (PVR) was 6.8 ± 4.2 Wood units. Indexed right-atrial volume averaged 55.2 ± 30.5 mL/m², and mean right-atrial pressure was 7.2 ± 4.6 mmHg. No significant baseline hemodynamic or echocardiographic differences were observed between surgical and medical groups.

**Table 2:** Echocardiographic and hemodynamic parameters at baseline.

Echocardiographic parameters, mean ± SD	All patients (*N* = 106)	PEA patients (*N* = 55)	Medical management (*N* = 51)	*p*-value
Right ventricular systolic pressure, mmHg	73.4 ± 26.8	77.2 ± 23.0	69.1 ± 29.4	0.121
Right atrium volume, indexed, mL/m^2^	55.2 ± 30.5	52.0 ± 23.3	58.9 ± 35.9	0.261
Right atrium size, cm^2^	25.5 ± 8.8	24.8 ± 7.4	26.1 ± 9.8	0.446
RHC hemodynamic parameters, mean ± SD	All patients (*N* = 82)	PEA patients (*N* = 49)	Medical management (*N* = 33)	*p*-value
Mean pulmonary artery pressure, mmHg	40.5 ± 14.3	40.0 ± 12.4	41.2 ± 16.6	0.704
Pulmonary vascular resistance, Wood units	6.8 ± 4.2	7.15 ± 4.40	6.31 ± 3.99	0.400
Right atrial pressure, mmHg	7.2 ± 4.6	6.8 ± 3.6	7.8 ± 5.6	0.321
Mixed venous oxygen saturation (SvO2), %	62.5 ± 10.4	61.4 ± 12.2	64.1 ± 7.7	0.254

PEA, pulmonary endarterectomy; RHC, right-heart catheterization.

### Pharmacologic treatment

Pharmacologic treatment patterns are summarized in Supplementary Table S1. Anticoagulation was used in 95.5% of patients overall, including all surgical patients and 90.7% of medically treated patients (*p* = 0.02). Vitamin K antagonists predominated in the surgical group (80.4%), whereas non-vitamin K antagonists were more commonly prescribed in the medical group (85.7%; both *p* < 0.001).

At the first evaluation, 36.4% of patients were not receiving pulmonary arterial hypertension (PAH)-targeted therapy, with a higher proportion among PEA patients (57.1% vs. 14.8%; *p* < 0.001). Riociguat use was higher among medically managed patients (66.7% vs. 21.4%; *p* < 0.001), and endothelin-receptor antagonists were also more frequently prescribed (16.7% vs. 3.6%; *p* = 0.022). Prostacyclin analogues were used infrequently in both groups.

### Hemodynamic and functional improvement after PEA

Among patients with available paired data, PEA was associated with significant improvements in pulmonary hemodynamics, right-heart structure, and functional capacity (Table 3). Mean pulmonary artery pressure decreased from 39.4 to 23.8 mmHg (Δ −15.6, *p* < 0.001), and pulmonary vascular resistance decreased from 7.97 to 3.86 Wood units (Δ −4.12, *p* = 0.008). Right ventricular systolic pressure and tricuspid regurgitation gradient declined substantially (both *p* < 0.001), accompanied by a reduction in pulmonary artery diameter and marked reverse remodeling of the right atrium, including significant decreases in indexed right atrial volume and area (both *p* < 0.001).

**Table 3:** Dynamic changes in clinical, echocardiographic, and hemodynamic parameters before and after pulmonary endarterectomy.

Variable	Pulmonary endarterectomy patients with paired pre- and post-operative data (*N* = 22)				
	Before PEA	After PEA	Mean change (Δ)	95% CI of change	*p*-value
Right heart catheterization parameters					
Mean pulmonary artery pressure, mmHg	39.4	23.8	−15.6	(−21.7, −9.4)	<0.001
Pulmonary vascular resistance, Wood units	7.97	3.86	−4.12	(−7.0, −1.2)	0.008
Mixed venous oxygen saturation (SvO2), %	61.6	69.2	+7.55	(5.1, 10.0)	<0.001
CO, Fick, L/min	4.42	4.98	+0.55	(−1.12, 0.01)	0.053
CI, L/min/m²	2.49	2.72	+0.23	(−0.54, 0.08)	0.135
Echocardiographic parameters					
TAPSE, cm	1.60	1.60	0.00	(−0.25, 0.25)	0.993
Right ventricular systolic pressure, mmHg	80.1	37.0	−43.1	(−55.1, −31.2)	<0.001
Right ventricular–pulmonary artery coupling ratio (TAPSE/sPAP)	0.24	0.50	+0.26	(0.15, 0.39)	<0.001
TR gradient, mmHg	69.4	28.1	−41.3	(−51.7, −30.9)	<0.001
Pulmonary artery diameter, cm	3.00	2.29	−0.71	(−1.01, −0.40)	<0.001
Right atrium volume, indexed, mL/m^2^	53.6	28.1	−25.5	(−35.6, −15.4)	<0.001
Right atrium size, cm	24.4	16.9	−7.4	(−11.0, −3.9)	<0.001
Clinical parameters					
6 min walking distance, m	214.3	371.2	+156.9	(76.7, 237.1)	0.002
NT-proBNP, pg/mL	2,305	605	−1,700	(−2,847, −554)	0.006

NT-proBNP, N-terminal pro–B-type natriuretic peptide; WU, wood units; CI, confidence interval; TAPSE/sPAP, tricuspid annular plane systolic excursion to systolic pulmonary artery pressure ratio. Mean change (Δ) represents the difference between post- and pre-PEA values (After minus Before).

Mixed venous oxygen saturation improved significantly (+7.55%, *p* < 0.001), while cardiac output demonstrated a borderline increase, and cardiac index remained stable. The right ventricular–pulmonary artery coupling ratio (TAPSE/sPAP) improved significantly (Δ + 0.26, *p* < 0.001), whereas conventional TAPSE values remained unchanged. Functional capacity improved substantially, with six-minute walk distance increasing by 157 m (*p* = 0.002) and NT-proBNP levels decreasing significantly (*p* = 0.006).

Overall, these findings demonstrate significant postoperative improvement in hemodynamic, structural, and functional parameters following PEA.

### Survival analysis and predictors of mortality

During a median follow-up of 39 months, 13 deaths occurred: 5 (8.9%) among PEA patients and 8 (14.8%) among medically treated patients. Twelve patients (10.9%) were lost to follow-up. Patients lost to follow-up were censored at the date of last known contact. Kaplan–Meier analysis demonstrated numerically higher cumulative survival in the surgical group throughout follow-up (91% in the PEA group vs. 85% in the medical group), although the difference was not statistically significant (log-rank *p* = 0.24). Median survival was not reached in either group. The overall 3-year survival rate for the entire cohort was consistent with outcomes reported in contemporary international registries.

On univariable Cox regression analysis, female sex (HR 0.33, 95% CI 0.11–0.98, *p* = 0.046) and higher mean pulmonary artery pressure (HR 1.07, 95% CI 1.02–1.12, *p* = 0.008) were associated with mortality. Higher estimated glomerular filtration rate showed a borderline inverse association with mortality (HR 0.97, 95% CI 0.95–1.00, *p* = 0.04) (Supplementary Table S2).

### Epidemiologic distribution and PEA trends

**Figure 1:**
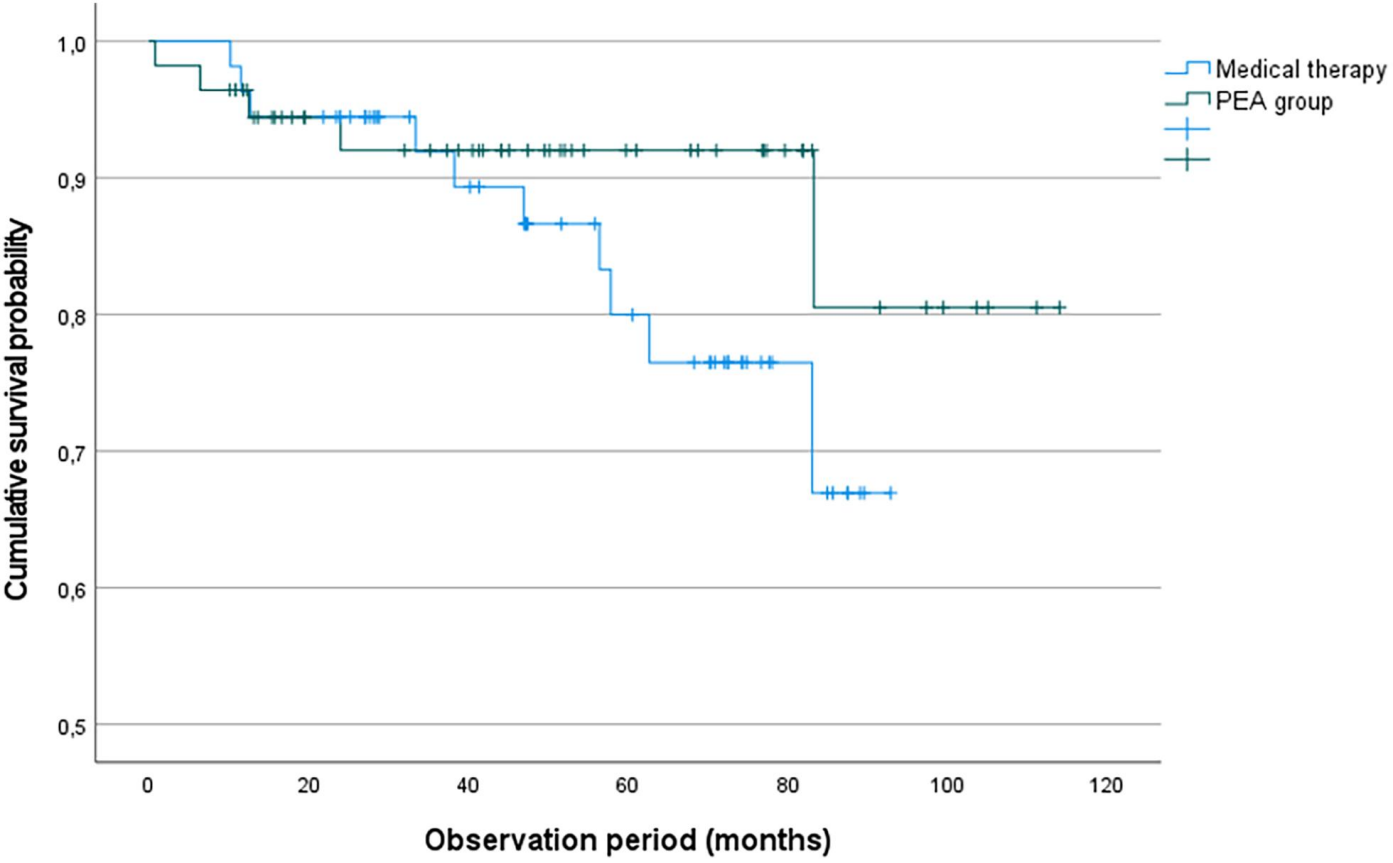
Kaplan–Meier survival curves comparing patients with chronic thromboembolic pulmonary hypertension treated with pulmonary endarterectomy (PEA) vs. medical therapy alone. Cumulative survival appeared numerically higher in the surgical group throughout follow-up, although the difference did not reach statistical significance (log-rank *p* = 0.24). Tick marks indicate censored observations.

Between 2018 and 2024, the annual nationally diagnosed catheter-confirmed CTEPH detection rate in Kazakhstan ranged from 0.38 to 1.52 cases per million population (pmp), with a mean of 0.83 pmp (Figure 1). Over the same period, the PEA rate increased from 0.22 pmp in 2018 to 0.70 pmp in 2024, with an average of 0.37 pmp. These values represent referral-based, catheter-confirmed cases rather than true population incidence and likely underestimate the true national burden of disease.

The annual PEA rate varied year to year, ranging from 0.05 to 0.70 pmp (Supplementary Figure S2). Despite these fluctuations, a progressive increase was observed toward the end of the study period, paralleling the national increase in CTEPH detection.

## Discussion

This study provides the first comprehensive national analysis of patients with chronic thromboembolic pulmonary hypertension (CTEPH) managed at a tertiary referral center in Kazakhstan, encompassing both surgically and medically treated populations over a seven-year period. The findings indicate that clinical characteristics, hemodynamic severity, and comorbidity profiles in this Central Asian cohort fall within the range reported in international registries, while highlighting regional challenges related to diagnostic delay, referral patterns, and treatment allocation. PEA was associated with substantial and durable improvements in pulmonary hemodynamics, right-heart structure, and functional capacity. The proportion of patients with residual pulmonary hypertension (23.8%) falls within the range reported in international surgical series.

At presentation, patients in this cohort were younger than those reported in recent international registries, with a mean age of 54 years and a near-equal sex distribution. In comparison, the Worldwide CTEPH Registry reported a mean age of 63 years and a lower proportion of male patients (7). Despite this younger demographic, the median interval of 24 months between symptom onset and referral to a specialized pulmonary hypertension center underscores a significant diagnostic delay. This exceeds the median diagnostic delay of 14–15 months reported in European and international cohorts (2, 7), emphasizing the ongoing challenge of delayed recognition of CTEPH and the need for structured post–PE surveillance and earlier referral of patients with unexplained dyspnea. A history of venous thromboembolism was common, with 69% of patients reporting prior PE and 56% deep-vein thrombosis. These proportions are comparable to those observed in the International CTEPH Registry and more recent global datasets (2, 7). In contrast, several Asian registries report lower rates of documented venous thromboembolism, likely reflecting under-recognition or differences in diagnostic practices (8, 9). These findings are compatible with the established two-hit model of CTEPH pathogenesis, in which persistent mechanical obstruction and abnormal vascular remodeling together drive disease progression (1, 10).

Most patients were classified as WHO functional class III, indicating advanced symptomatic limitation, and had San Diego classification levels I-II, reflecting the anatomical extent of thromboembolic disease. The mean six-minute walk distance of 265 ± 95 m reflected substantial functional limitation and did not differ significantly between surgical and medically managed patients, consistent with international cohorts reporting baseline distances of approximately 300 ± 120 m (2, 7). This likely reflects late diagnosis and referral bias, whereby both operable and inoperable patients present with similarly advanced symptomatic burden. Body mass index was higher among medically managed patients (30 ± 6.4 vs. 27.1 ± 4.1 kg/m^2^; *p* = 0.004). However, current surgical series have not demonstrated an adverse effect of obesity on PEA outcomes (11), suggesting BMI alone is unlikely to determine operability.

Baseline echocardiographic and right-heart catheterization parameters demonstrated advanced pulmonary hypertension, with mean pulmonary artery pressure around 40 ± 14.3 mmHg and pulmonary vascular resistance approximately 6.8 ± 4.2 Wood units, without significant difference between surgical and medically managed patients. These values are broadly consistent with those reported in the large International Registry, the US-CTEPH Registry, and the Japanese nationwide CTEPH Registry (7, 8, 12). Importantly, no significant baseline hemodynamic differences were observed between patients selected for PEA and those managed medically, indicating that operability decisions were primarily influenced by the anatomic distribution of thromboembolic lesions rather than by hemodynamic severity, consistent with current international practice (1, 13).

Medical therapy played a central role in patients with inoperable or residual CTEPH. Nearly all patients received lifelong anticoagulation, consistent with contemporary guideline recommendations and international registry data (1). A transition toward non–vitamin K oral anticoagulants was observed, reflecting evolving global practice patterns and emerging evidence of their use in the selected CTEPH population (7). PAH-targeted therapies were widely used in medically managed patients, with 85.2% receiving at least one agent, most commonly riociguat (66.7%), in accordance with contemporary ESC/ERS guideline recommendations (1).

PEA resulted in marked hemodynamic and functional improvement, with mPAP decreasing by 16 mmHg and PVR declining by approximately 52%, accompanied by a meaningful increase in 6-minute walk distance of nearly 160 m. These outcomes closely align with reports from high-volume international centers, where postoperative reductions in pulmonary vascular resistance typically range from 55% to 65% and sustained functional improvement is observed in the majority of patients (7, 8, 12). The favorable postoperative outcomes observed in this cohort suggest that outcomes within the ranges reported by established international centers are achievable within a newly established national CTEPH program.

In our cohort, three-year survival following PEA (92%) was similar to that reported in the contemporary worldwide CTEPH registry (94%) and other high-volume surgical series, including Dardi et al. (7, 14). Although survival numerically favored surgical treatment, the difference compared with medical therapy alone did not reach statistical significance (log-rank *p* = 0.24), likely reflecting limited statistical power. Survival analyses should therefore be interpreted as exploratory and hypothesis-generating, given the limited number of events and incomplete follow-up. In univariable analysis, higher mean pulmonary artery pressure and impaired renal function were associated with increased mortality, consistent with prior studies identifying postoperative hemodynamic severity as a key determinant of long-term outcome after PEA (15) and demonstrating that reduced glomerular filtration rate (GFR) is associated with adverse clinical outcomes in CTEPH populations (16). Female sex showed a protective association in univariable analysis; however, these findings should be interpreted cautiously, given the limited number of events. In the European CTEPH registry, women underwent PEA less frequently than men but demonstrated improved long-term survival (adjusted hazard ratio 0.55), while short-term mortality was similar between the sexes (17).

At the population level, the nationally recorded catheter-confirmed CTEPH case detection rates in Kazakhstan were substantially lower than those reported in international registries, as was the national PEA rate (7). Contemporary data indicate that the cumulative diagnosis of CTEPH following acute pulmonary embolism approaches approximately 2%–3% at 3 years in routine clinical practice (18). These figures likely underestimate the true burden in Kazakhstan, where diagnostic delays, the absence of structured post-PE surveillance, and limited referral pathways may reduce case detection. Limited availability of ventilation-perfusion (V/Q) scintigraphy—the guideline-recommended screening modality with high sensitivity for CTEPH, may further contribute to under-recognition (1, 19). Similar diagnostic and referral challenges have been described in non-Western and middle-income settings (20). Marked regional variation in detected prevalence further supports the role of referral patterns and diagnostic capture in shaping observed disease burden.

Strengths of this study include comprehensive national case inclusion over an extended period, detailed hemodynamic characterization, and paired pre- and post-PEA analyses using standardized outcome measures. Limitations include the retrospective single-center design and potential referral bias. Importantly, anatomical operability descriptors (e.g., proximal vs. distal disease, surgical level classification) were not systematically captured in the registry. Consequently, baseline comparability between surgical and medically treated groups cannot be fully established, and between-group differences should not be interpreted as causal treatment effects. Incomplete follow-up for certain parameters and limited statistical power for survival analyses further restrict definitive inference. The small number of mortality events limited the ability to perform multivariable survival modeling; therefore, survival predictors should be interpreted as exploratory. Only 22 of 56 surgical patients had complete paired follow-up data. This subgroup comprises patients with available postoperative reassessment and may not fully represent the surgical cohort, potentially introducing selection bias into the estimation of treatment effects. The absence of balloon pulmonary angioplasty during the study period limits comparison with contemporary multimodal treatment strategies available in high-volume international centers.

In conclusion, this national experience indicates that PEA provides substantial hemodynamic and clinical benefit in appropriately selected patients with CTEPH, with outcomes falling within ranges reported by established international centers. Optimized medical therapy offers reasonable long-term outcomes for patients with inoperable disease. These findings highlight the need for earlier diagnosis, structured post–pulmonary embolism follow-up, and continued development of multidisciplinary CTEPH programs to improve outcomes in emerging healthcare systems.

## Data Availability

The raw data supporting the conclusions of this article will be made available by the authors, without undue reservation.

## Supplementary material

Supplementary FIgure S1 Flow diagram of patient selection and study cohort formation. Discharge summaries were screened to identify patients hospitalized with a principal diagnosis of chronic thromboembolic pulmonary hypertension (CTEPH). After exclusion of repeat admissions, unique patients were classified according to treatment strategy (medical therapy only or pulmonary endarterectomy). A subset of surgical patients with follow-up admissions was analyzed for post-endarterectomy outcomes, and all patients were included in the survival analysis.

Supplementary FIgure S2 Annual nationally diagnosed catheter-confirmed CTEPH detection rate and pulmonary endarterectomy (PEA) rates per million population (pmp) in Kazakhstan from 2018 to 2024. The national detection rate for CTEPH remained low overall, whereas the number of surgical procedures increased over time, reflecting the expansion of national referral pathways and surgical capacity.

## References

[1] Humbert M Kovacs G Hoeper MM Badagliacca R Berger RMF Brida M 2022 ESC/ERS guidelines for the diagnosis and treatment of pulmonary hypertension. Eur Heart J. (2022) 43(38):3618–731. 10.1093/eurheartj/ehac237 36017548

[2] Pepke-Zaba J Delcroix M Lang I Mayer E Jansa P Ambroz D Chronic thromboembolic pulmonary hypertension: results from an international prospective registry. Circulation. (2011) 124(18):1973–81. 10.1161/CIRCULATIONAHA.110.015008 21969018

[3] Lang IM Madani MM. Update on chronic thromboembolic pulmonary hypertension. Circulation. (2014) 130(6):508–18. 10.1161/CIRCULATIONAHA.114.009309 25092279

[4] Mizoguchi H Ogawa A Munemasa M Mikouchi H Ito H Matsubara H. Refined balloon pulmonary angioplasty for inoperable patients with chronic thromboembolic pulmonary hypertension. Circulation. (2012) 126(7):850–9. 10.1161/CIRCULATIONAHA.112.104463 23192917

[5] Ghofrani HA D'Armini AM Grimminger F Hoeper MM Jansa P Kim NH Riociguat for the treatment of chronic thromboembolic pulmonary hypertension. N Engl J Med. (2013) 369(4):319–29. 10.1056/NEJMoa1209657 23883377

[6] Leber L Beaudet A Muller A. Epidemiology of pulmonary arterial hypertension and chronic thromboembolic pulmonary hypertension: identification of the most accurate estimates from a systematic literature review. Pulm Circ. (2021) 11(1):2045894020977300. 10.1177/2045894020977300 33456755 PMC7797595

[7] Delcroix M Torbicki A Gopalan D Sitbon O Klok FA Lang IM Worldwide CTEPH registry: long-term outcomes and treatment patterns. Circulation. (2024) 149:135–48. 10.1161/CIRCULATIONAHA.123.06734538084582

[8] Masaki K Hosokawa K Funakoshi K Taniguchi Y Adachi S Inami T Outcomes of chronic thromboembolic pulmonary hypertension after balloon pulmonary angioplasty and pulmonary endarterectomy. JACC Asia. (2024) 4(8):577–89. 10.1016/j.jacasi.2024.05.007 39156509 PMC11328766

[9] Wang KL Yap ES Goto S Zhang S Siu CW Chiang CE. The diagnosis and treatment of venous thromboembolism in Asian patients. Thromb J. (2018) 16:4. 10.1186/s12959-017-0155-z 29375274 PMC5774147

[10] Biondi F Alberti M Montemaggi E D'Alleva A Madonna R. Not just CTEPH: a narrative review on the spectrum approach to post-pulmonary embolism conditions. Thromb Haemost. (2025) 125(7):634–42. 10.1055/a-2418-7895 39299271

[11] Fernandes TM Auger WR Fedullo PF Kim NH Poch DS Madani MM Baseline body mass index does not significantly affect outcomes after pulmonary thromboendarterectomy. Ann Thorac Surg. (2014) 98(5):1776–81. 10.1016/j.athoracsur.2014.06.045 25240778 PMC4254364

[12] Kerr KM Elliott CG Chin K Benza RL Channick RN Davis RD Results from the United States chronic thromboembolic pulmonary hypertension registry: enrollment characteristics and 1-year follow-up. Chest. (2021) 160(5):1822–31. 10.1016/j.chest.2021.05.052 34090871 PMC8628169

[13] Jenkins DP Biederman A D'Armini AM Dartevelle PG Gan HL Klepetko W Operability assessment in CTEPH: lessons from the CHEST-1 study. J Thorac Cardiovasc Surg. (2016) 152(3):669–674.e3. 10.1016/j.jtcvs.2016.02.062 27083940

[14] Dardi F Manes A Guarino D Suarez SM Loforte A Rotunno M Long-term outcomes after pulmonary endarterectomy. Ann Cardiothorac Surg. (2022) 11(2):172–4. 10.21037/acs-2021-pte-179 35433360 PMC9012203

[15] Cannon JE Su L Kiely DG Page K Toshner M Swietlik E Dynamic risk stratification of patient long-term outcome after pulmonary endarterectomy: results from the United Kingdom national cohort. Circulation. (2016) 133(18):1761–71. 10.1161/CIRCULATIONAHA.115.019470 27052413 PMC5860739

[16] Chakinala MM Coyne DW Benza RL Frost AE Farber HW Elliott CG Impact of declining renal function on outcomes in pulmonary arterial hypertension: a REVEAL registry analysis. J Heart Lung Transplant. (2018) 37(6):696–705. 10.1016/j.healun.2018.02.012 29174533

[17] Barco S Klok FA Konstantinides SV Dartevelle P Fadel E Jenkins D Sex-specific differences in chronic thromboembolic pulmonary hypertension: results from the European CTEPH registry. J Thromb Haemost. (2020) 18(1):151–61. 10.1111/jth.14629 31479557

[18] Ikeda N Yamashita Y Morimoto T Chatani R Kaneda K Nishimoto Y Incidence of chronic thromboembolic pulmonary hypertension after pulmonary embolism in the era of direct oral anticoagulants: from the COMMAND VTE registry-2. J Am Heart Assoc. (2024) 13(21):e035997. 10.1161/JAHA.124.035997 39435728 PMC11935678

[19] Tunariu N Gibbs SJ Win Z Gin-Sing W Graham A Gishen P Ventilation-perfusion scintigraphy is more sensitive than multidetector CTPA in detecting chronic thromboembolic pulmonary disease as a treatable cause of pulmonary hypertension. J Nucl Med. (2007) 48(5):680–4. 10.2967/jnumed.106.039438 17475953

[20] Dzudie A Dzekem BS Ojji DB Kengne AP Mocumbi AO Sliwa K Pulmonary hypertension in low- and middle-income countries with focus on sub-saharan Africa. Cardiovasc Diagn Ther. (2020) 10(2):316–24. 10.21037/cdt.2019.07.06 32420114 PMC7225434

