# Supplementary material for: Clinical characteristics, management strategies, and survival outcomes of patients with chronic thromboembolic pulmonary hypertension in Central Asia: experience from the sole pulmonary endarterectomy center
Source: Front Cardiovasc Med. 2026 Mar 24;13:1786958. doi: 10.3389/fcvm.2026.1786958 (PMC13054472; doi:10.3389/fcvm.2026.1786958)
Supplement: Supplementary file 1 [file Table1.docx]

**Clinical characteristics, management strategies, and survival outcomes of patients with chronic thromboembolic pulmonary hypertension in Central Asia: experience from the sole pulmonary endarterectomy center**

**Authors:** Anara Abbay ^1^*, Akbota Askanbekova^1^, Yuliya Semenova^1^, Aigerim Kuzhakhmetova^2^, Gulzhamal Duysenbay^2^, Murat Mukarov^2^, Timur Lesbekov^2^

**Affiliations:**

^1^ Department of Medicine, Nazarbayev University School of Medicine, Astana, Kazakhstan

^2^ Department of Cardiac Surgery, Heart Center, University Medical Center, Astana, Kazakhstan

Anara Abbay and Akbota Askanbekova contributed equally to this article

**Corresponding author:** Anara Abbay, email address: [anara.abbay@nu.edu.kz](mailto:anara.abbay@nu.edu.kz)

**Supplementary Table 1.** Pharmacologic treatment at baseline

| **Anticoagulation, n (%)** | **All patients** | **PEA patients** | **Medical management** | **p-value** |
| --- | --- | --- | --- | --- |
| Patients receiving anticoagulation, n (%) | 105 (95.5) | 56 (100.0) | 49 (90.7) | 0.020 |
| Vitamin K antagonists | 52 (49.5) | 45 (80.4) | 7 (14.3) | <0.001 |
| Novel oral anticoagulant | 53 (50.5) | 11 (19.6) | 42 (85.7) | <0.001 |
| **PAH medications at diagnosis or first hospital visit, n (%)** |  |  |  |  |
| No PAH therapy | 40 (36.4%) | 32 (57.1%) | 8 (14.8%) | <0.001 |
| 1-component therapy | 52 (47.3%) | 18 (32.1%) | 34 (63.0%) |  |
| 2-component therapy | 12 (10.9%) | 5 (8.9%) | 7 (13.0%) |  |
| 3-component therapy | 6 (5.5%) | 1 (1.8%) | 5 (9.3%) |  |
| **PAH medications** |  |  |  |  |
| Soluble guanylate cyclase stimulator | 48 (43.6) | 12 (21.4) | 36 (66.7) | <0.001 |
| Phosphodiesterase type inhibitor | 26 (23.6) | 15 (26.8) | 11 (20.4) | 0.429 |
| Endothelin receptor antagonist | 11 (10.0) | 2 (3.6) | 9 (16.7) | 0.022 |
| Prostacyclin analogue | 8 (7.3) | 2 (3.6) | 6 (11.1) | 0.128 |

PEA – Pulmonary endarterectomy; PAH – Pulmonary arterial hypertension; SD – Standard deviation; WU – Wood units
