# Supplementary material for: Clinical characteristics, management strategies, and survival outcomes of patients with chronic thromboembolic pulmonary hypertension in Central Asia: experience from the sole pulmonary endarterectomy center
Source: Front Cardiovasc Med. 2026 Mar 24;13:1786958. doi: 10.3389/fcvm.2026.1786958 (PMC13054472; doi:10.3389/fcvm.2026.1786958)
Supplement: Supplementary file 2 [file Table2.docx]

**Clinical characteristics, management strategies, and survival outcomes of patients with chronic thromboembolic pulmonary hypertension in Central Asia: experience from the sole pulmonary endarterectomy center**

**Authors:** Anara Abbay ^1^*, Akbota Askanbekova^1^, Yuliya Semenova^1^, Aigerim Kuzhakhmetova^2^, Gulzhamal Duysenbay^2^, Murat Mukarov^2^, Timur Lesbekov^2^

**Affiliations:**

^1^ Department of Medicine, Nazarbayev University School of Medicine, Astana, Kazakhstan

^2^ Department of Cardiac Surgery, Heart Center, University Medical Center, Astana, Kazakhstan

Anara Abbay and Akbota Askanbekova contributed equally to this article

**Corresponding author:** Anara Abbay, email address: [anara.abbay@nu.edu.kz](mailto:anara.abbay@nu.edu.kz)

# Supplementary Table 2. Univariable Cox regression analyses of predictors of mortality in patients with CTEPH

| **Variable** | **HR (95% CI)** | **p-value** |
| --- | --- | --- |
| Age (per year) | 1.02 (0.97-1.06) | 0.48 |
| Gender (female) | 0.33 (0.11-0.98) | 0.046 |
| Pulmonary endarterectomy | 0.53 (0.18-1.55) | 0.25 |
| Pulmonary embolism | 0.15 (0.02-1.25) | 0.08 |
| Deep vein thrombosis | 1.23 (0.43-3.49) | 0.70 |
| Varicose veins | 2.16 (0.68-6.85) | 0.19 |
| Body mass index (per kg/m²) | 1.07 (0.998-1.15) | 0.056 |
| Right ventricular systolic pressure (mmHg) | 1.01 (0.99-1.03) | 0.28 |
| Mean pulmonary artery pressure (mmHg) | 1.07 (1.02-1.12) | 0.008 |
| Pulmonary vascular resistance (per WU) | 1.07 (0.91-1.26) | 0.39 |
| N-terminal pro-B-type natriuretic peptide (per pg/mL) | 1.00 (1.00-1.00) | 0.40 |
| Estimated glomerular filtration rate (per 1 mL/min/1.73 m²) | 0.97 (0.95-1.00) | 0.04 |

**WU** – Wood units; CI – confidence interval; HR – hazard ratio
