# Supplementary figures and images for: Clinical characteristics, management strategies, and survival outcomes of patients with chronic thromboembolic pulmonary hypertension in Central Asia: experience from the sole pulmonary endarterectomy center
Source: Front Cardiovasc Med. 2026 Mar 24;13:1786958. doi: 10.3389/fcvm.2026.1786958 (PMC13054472; doi:10.3389/fcvm.2026.1786958)

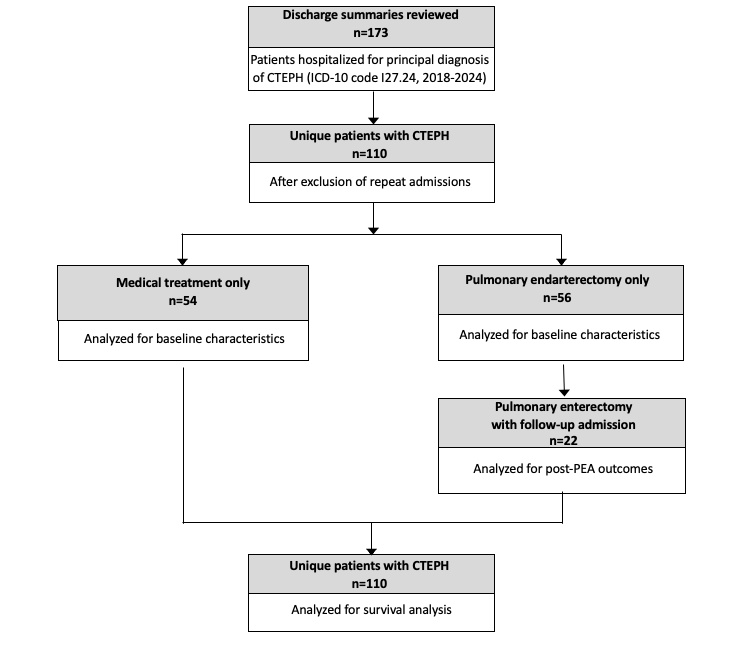

Supplement: Supplementary FIgure S1 — Flow diagram of patient selection and study cohort formation. Discharge summaries were screened to identify patients hospitalized with a principal diagnosis of chronic thromboembolic pulmonary hypertension (CTEPH). After exclusion of repeat admissions, unique patients were classified according to treatment strategy (medical therapy only or pulmonary endarterectomy). A subset of surgical patients with follow-up admissions was analyzed for post-endarterectomy outcomes, and all patients were included in the survival analysis. [file Image1.jpeg]

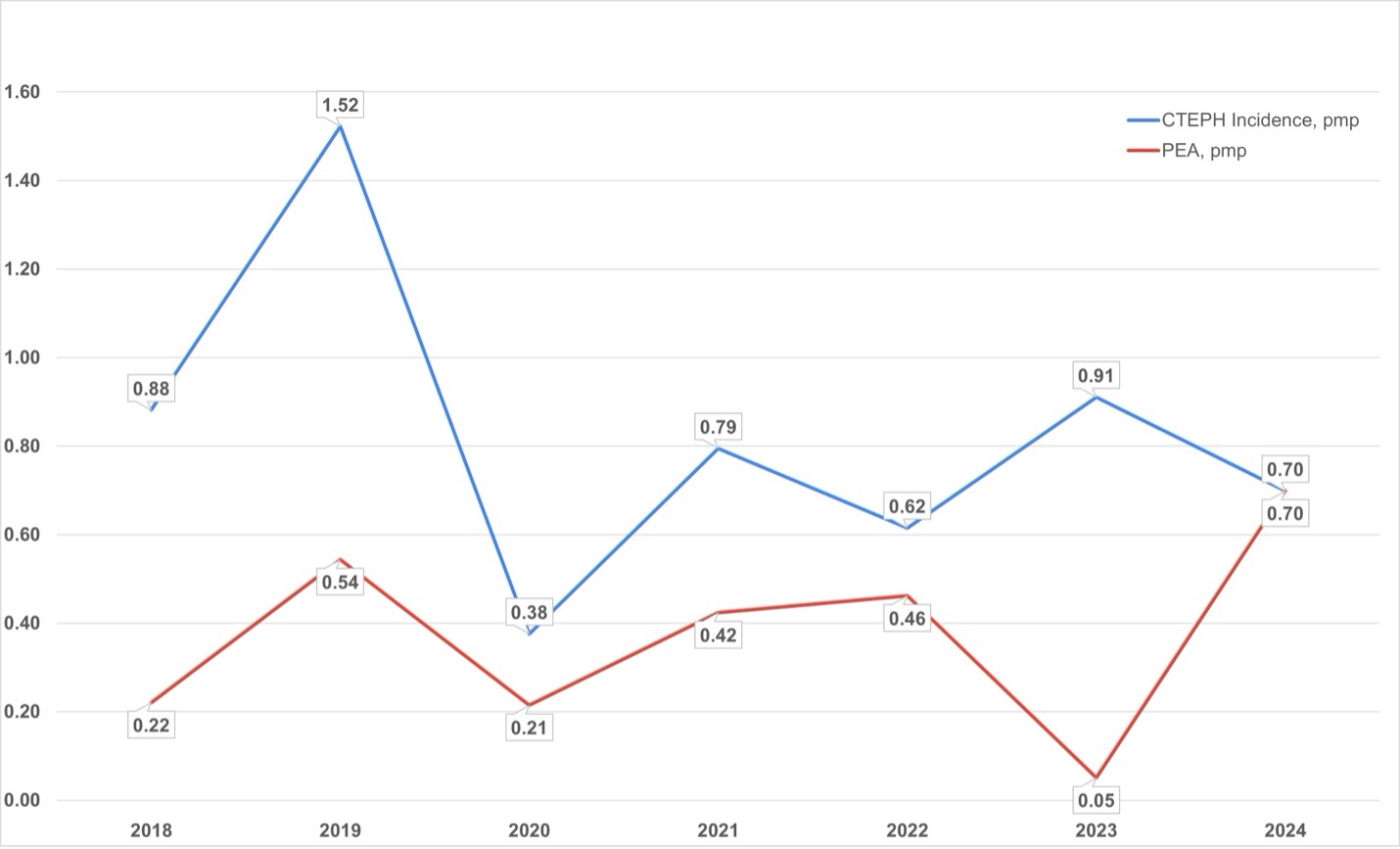

Supplement: Supplementary FIgure S2 — Annual nationally diagnosed catheter-confirmed CTEPH detection rate and pulmonary endarterectomy (PEA) rates per million population (pmp) in Kazakhstan from 2018 to 2024. The national detection rate for CTEPH remained low overall, whereas the number of surgical procedures increased over time, reflecting the expansion of national referral pathways and surgical capacity. [file Image2.jpeg]
